# Young Adults with High Autistic-Like Traits Displayed Lower Food Variety and Diet Quality in Childhood

**DOI:** 10.1007/s10803-020-04567-4

**Published:** 2020-07-02

**Authors:** Catherine Panossian, Philippa Lyons-Wall, Andrew Whitehouse, Wendy H. Oddy, Johnny Lo, Jane Scott, Therese A. O’Sullivan

**Affiliations:** 1 School of Medical and Health Sciences, Edith Cowan University, 270 Joondalup Drive, Joondalup, WA 6027 Australia; 2 Telethon Kids Institute, The University of Western Australia, Perth Children’s Hospital, Northern Entrance, 15 Hospital Avenue, Nedlands, WA 6009 Australia; 3 Menzies Institute for Medical Research, University of Tasmania, Churchill Avenue, Hobart, TAS 7005 Australia; 4 School of Science, Edith Cowan University, 270 Joondalup Drive, Joondalup, WA 6027 Australia; 5 School of Public Health, Curtin University, Kent Street, Bentley, WA 6102 Australia

**Keywords:** Autistic-like traits, Autism spectrum disorder, Food variety, Diet quality, Child, Young adult

## Abstract

This study explored the association between autistic-like traits in young adults and dietary intake in early childhood in the Gen2 Raine Study cohort. Data were available from 811 participants at years 1, 2 and 3 for the assessment of dietary intake, and at year 20 for measurement of autistic-like traits. Results showed as autistic-like traits increased, total food variety, core food variety and dairy variety decreased (p < 0.05), with a lower consumption of citrus fruits and yoghurt (both p = 0.04). As autistic-like traits increased, diet quality decreased, this trend was significant at 2 years (p = 0.024). Our results suggest that young adults with higher autistic-like traits were more likely to have had lower food variety and diet quality in early childhood.

**Electronic supplementary material:**

The online version of this article (10.1007/s10803-020-04567-4) contains supplementary material, which is available to authorized users.

## Introduction

Autism Spectrum Disorder (ASD) is a developmental disability characterised by deficits in social and communication interactions, and the presence of restricted and repetitive displays of interests, behaviours or activities (American Psychiatric Association 2013). There is increasing evidence that the traits of ASD sit on a continuum within the general population, with the extreme end of the distribution representing clinical ASD (Whitehouse et al. 2011). Some cognitive and behavioural difficulties that people with ASD experience—such as social skills, attention switching, communication, imagination and attention to details (Baron-Cohen et al. 2001)—can also be observed in those without clinical ASD, usually to a milder degree or below the threshold of an ASD diagnosis (Clark et al. 2013; Khanjani et al. 2018; Nakamura et al. 2019). There is a growing appreciation that understanding factors that are associated with autistic-like traits within the general population could help provide further insights into clinical ASD (Whitehouse et al. 2011).

Children with ASD have an estimated fivefold increase in the prevalence of feeding problems compared to typically developing children (Sharp et al. 2013). Common feeding problems in children with ASD include restrictive and selective food intake; food neophobia (fear of trying new foods); increased sensory sensitivity; and pica (eating non-edible foods) (Ranjan and Nasser 2015). Feeding difficulties in children with ASD may also pose a substantial health risk (Cermak et al. 2010). In severe cases, there have been reports of scurvy, vision loss, rickets (Sharp et al. 2018), low bone mineral density (Hediger et al. 2008; Neumeyer et al. 2018), malnutrition and growth retardation (Sharp et al. 2013).

Low food variety has been frequently noted in studies that have investigated feeding difficulties in children with ASD (Attlee et al. 2015; Bicer and Alsaffar 2013; Kral et al. 2013; Marshall et al. 2014; Nadon et al. 2011; Ranjan and Nasser 2015). A diet low in food variety has been shown to impact the quality of the person’s diet (Kral et al. 2013), which raises concerns that the diet of some children with ASD may be nutritionally inadequate (Bandini et al. 2010; Cermak et al. 2010; Kral et al. 2013; Kuschner et al. 2015; Zimmer et al. 2012). Foods reported as lacking include: milk and other dairy products (Herndon et al. 2009; Marshall et al. 2014; Neumeyer et al. 2018; Schreck et al. 2004; Sharp et al. 2018); vegetables (Bandini et al. 2010; Chistol et al. 2018; Emond et al. 2010; Marshall et al. 2014; Ranjan and Nasser 2015; Schreck et al. 2004; Sharp et al. 2013); and fruit (Chistol et al. 2018; Emond et al. 2010; Marshall et al. 2014; Schreck et al. 2004). Conversely, other studies have reported a higher intake of energy dense foods (Diolordi et al. 2014; Ranjan and Nasser 2015), particularly those high in fat and sugar (Cermak et al. 2010).

Few studies have investigated feeding issues and autistic-like traits in the general population. A systematic review comprising seven studies showed that adolescents and adults with anorexia nervosa had significantly more autistic-like traits compared to controls (Westwood et al. 2016). A cross-sectional study in Japan showed that autistic-traits were associated with lower intakes of nutrients in the general adult population (Nakamura et al. 2019). Furthermore, a study among university students showed that an increase in autistic-like traits was associated with an increase in food neophobia, suggesting that those with higher autistic-like traits are less likely to eat new foods (Stafford et al. 2017). Similarly, Clark et al. (2013) showed that those with higher autistic-like traits (mean age: 22.6 years), found it more difficult to deal with conflicting sensory information, for example when drinks were presented as green in colour and strawberry in flavour. To our knowledge, only one study has explored autistic-like traits and eating behaviours among children. This population-based cohort in the Netherlands showed that autistic traits measured at six years, were associated with more emotional eating, picky eating and food responsiveness (response to sight and smell of food) at ten years of age (van 't Hof et al. 2020).

Our study was conducted in a prospective cohort of healthy young adults. The aim was to explore the association between autistic-like traits in young adults and food variety and diet quality in early childhood. Use of longitudinal data can provide unique information on the trajectory of health behaviours and outcomes. Feeding problems are highly prevalent in children with ASD, and we hypothesised that those with more autistic-like traits in adulthood had childhood diets that were lower in food variety and quality. We also investigated the consumption of specific food types in order to identify those foods that may be under or over consumed. As ASD lies on a spectrum and contains no fluid borders, it is important to research autistic-like traits in the general population to enhance understanding into clinical ASD. In addition, diet is an essential part of daily life, and research into its relationship with autistic-like traits is warranted. This information may help to inform dietary advice to support children and their families as they grow.

## Methods

### Participants

The participants were from the population-based Raine Study, details of which have been reported previously (Newnham et al. 1993). In summary, between May 1989 and November 1991, 2900 pregnant women of 16–20 weeks gestation were recruited from local private clinics and the main public antenatal clinics at King Edward Memorial Hospital for Women in Perth, Western Australia. A total of 2868 live births occurred, and these children have had comprehensive follow-up assessments at regular intervals. Information gathered has included questionnaire data, biological sample information and clinical assessment data. Requirements to enter the study included sufficient English to understand the assessments, and their intention to remain in Western Australia for future follow-up assessments. The use of this longitudinal study has allowed us to look back in time at the participant's food intake as a child and compare it to their degree of autistic-like traits as a young adult.

**Fig. 1:**
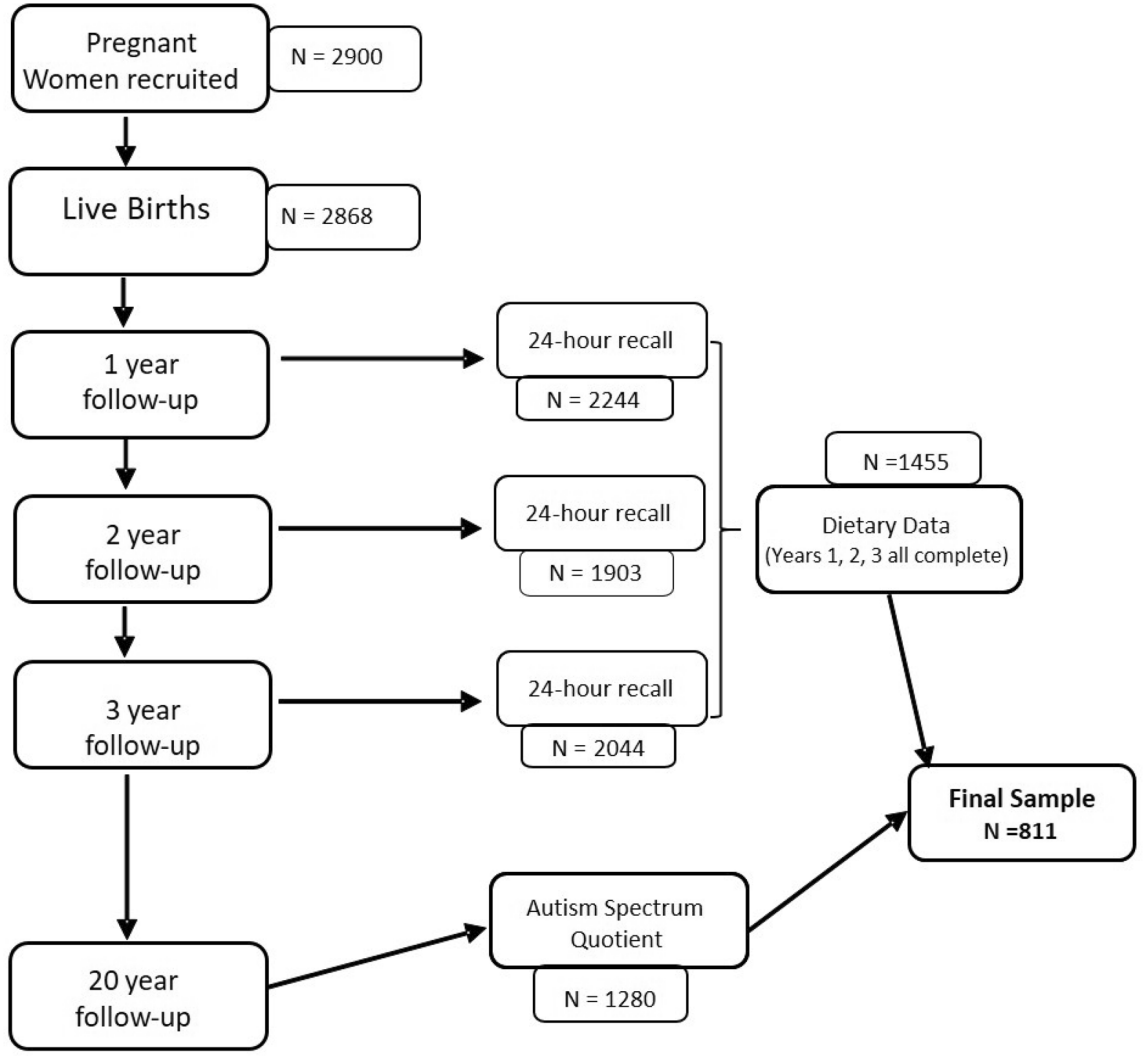
Study design flow chart for participants selected from the Raine Study

For the current study, data were utilised from the Raine Study (Gen2) participants, with follow-up assessments conducted at ages 1, 2, 3 years for dietary intake and age 20 years for autistic-like traits. Figure 1 displays a flow chart of the study design.

### Ethics

The protocol for the Raine Study was approved by the Human Ethics Committees of King Edward Memorial Hospital for Women and Princess Margaret Hospital for Children, in Perth Western Australia. An Ethics Declaration was also approved by the Edith Cowan University Human Research Ethics Committee.

### Dietary Assessment

Dietary data were collected from the primary caregivers at the 1, 2- and 3-year follow-ups, using a single 24-h recall. The primary caregiver was asked to supply the type and quantity of food and drinks consumed by the child over one day. Participants in this study must have completed all three 24-h recalls.

### Food Variety Score

The 24-h recall data were used to develop a Food Variety Score to represent the range of foods consumed at three time points over three years of follow-up (Scott et al. 2012). Food items were assigned into 1 of 40 food subgroups to investigate overall variety in the diet (Table 1). For the purpose of this study, food variety was defined as the number of different food types eaten daily, irrespective of frequency or quantity.

**Table 1:** Food groups used to develop the food variety scores

Food variety groups	Food variety sub-groups
Dairy Variety (Dairy & dairy alternatives)	Milk ^a^ Cheese Yoghurt ^b^ Milk desserts^†^^c^
Grain Variety (Grain & cereal products)	Unrefined breakfast cereals ^d^ Refined breakfast cereals ^e^ Breads and rolls Rice and pasta ^f^ Crackers ^g^ Cereal or granola bars^†^ Pizza/savoury pies and pastries^†^
Fruit & Vegetable Variety (all fruits and vegetables)	Cruciferous vegetables ^h^ Yellow/orange vegetables ^i^ Potatoes ^j^ Other vegetables ^k^ Green vegetables ^l^ Tomatoes Hot chips/french fries^†^
	Apples Bananas Citrus fruits Pears Summer fruits ^m^ Dried fruits Other fruits ^n^
Meat & Alternatives Variety (meat & meat alternatives)	Eggs Nuts and seeds ^o^ Legumes and other vegetarian substitutes ^p^ Red meat ^q^ Chicken or turkey Fish and shellfish Offal and unspecified meats Hotdogs, sausages and cold deli meats^†^^r^
Discretionary Variety (Non-core foods)^†^	Cakes, pies, cookies, sweet pastries Added sugar and other desserts ^s^ Confectionary ^t^ Carbonated soft drinks Fruit flavoured drinks ^u^ Salty snacks ^v^ Added fat ^w^

^†^Considered discretionary foods (not necessary to provide required nutrients and may be high in saturated fats, sugars or salt)

^a^Includes dairy, soy, breast, formula and flavoured milks

^b^Includes dairy and soy yoghurts

^c^Includes cream, ice-cream and custard

^d^Includes high-fibre breakfast cereals and wholegrains

^e^Includes refined grains and low-fibre breakfast cereals

^f^Includes rice cereal for infants

^g^Includes high-fibre and low-fibre crackers, pretzels, rice cakes and rusks

^h^Includes broccoli, cauliflower and cabbage

^i^Includes carrot, pumpkin and sweet potato

^j^Excludes French fries/hot chips

^k^Includes mushrooms, corn, other root vegetables, mixed vegetables, marrow and seaweed

^l^Includes leaf, stalk and other green vegetables

^m^Includes berries, melons and stone fruit

^n^Includes other fruits and mixed fruit salad

^o^Includes peanut butter

^p^Includes dried beans and lentils, fresh legumes (peas and beans), and vegetarian meat alternatives

^q^Includes beef, lamb, kangaroo and pork

^r^Includes ham

^s^Includes added sugar, syrups, preserves and other desserts

^t^Includes lollies, chocolate and ice confectionary

^u^Includes cordial, fruit juice and other fruit drinks

^v^Includes potato crisps, popcorn, corn chips and other chips and salty snacks

^w^Includes added fats and oils, sauces, dressings, dips and spreads

This table was adapted from the Food Variety Score developed by Scott et al. (2012)

A “0” was given to mark no intake, and a “1” was given if the food sub-group was eaten on one or more occasions that day. The total Food Variety Score had a maximum score of 40, with a higher score indicating higher variety in the diet. This total score was further divided into a Core Food Variety Score and a Discretionary Variety Score. The Core Food Variety Score had a maximum score of 28 and included healthy foods from the five core food groups: dairy and dairy alternatives, grain and cereal products, vegetables, fruits, and meat and meat alternatives (National Health and Medical Research Council 2013). The Core Food Variety Score was further divided into individual food groups including: a Fruit and Vegetable Variety Score with a maximum of 13, Dairy Variety Score with a maximum of 3; Grain Variety Score with a maximum of 5; and Meat and Alternatives Variety Score with a maximum of 7. The Discretionary Variety Score had a maximum score of 12 and included non-core foods that were energy-dense and nutrient-poor and typically high in fat, sugar and/or salt (National Health and Medical Research Council 2013).

### The Raine Eating Assessment in Toddlers Score

The Raine Eating Assessment in Toddlers (EAT) score was used to measure the diet quality of children at ages 1, 2 and 3 years. The EAT score took into consideration whether food and beverages consumed were healthy or discretionary foods. The EAT score was previously developed and scored, and details have been reported elsewhere (Meyerkort et al. 2012; Nyaradi et al. 2013). In summary, the EAT Score was comprised of seven food groups: (1) wholegrains, (2) vegetables, (3) fruit, (4) meat, (5) dairy, (6) snack foods, and (7) soda and drinks. The first 5 food groups considered healthy foods and scored out of 10; greater frequency of eating these foods gave them a score closer to 10. Food groups 6 and 7 were considered unhealthy or discretionary foods and greater frequency of consuming these foods gave them a score closer to zero. The total EAT score was calculated by adding all the seven food groups together and had a possible range from 0 to 70. A higher score indicated a higher quality diet, meaning that participants ate more healthy food and less unhealthy food. Breastmilk and infant formula were excluded from the EAT Score.

### Autism Spectrum Quotient

At the 20 year follow-up, participants from the Raine Study were asked to complete the Autism Spectrum Quotient (AQ), a self-report questionnaire designed to measure autistic-like traits within the general adult population (Baron-Cohen et al. 2001). Participants were asked to rate 50 statements on how well they applied to them on a four-point scale: definitely agree, slightly agree, slightly disagree or definitely disagree. An example of an autistic-like-trait is “I prefer to do things the same way over and over again”, or “I often notice small sounds when others do not” (Baron-Cohen et al. 2001). The Total Autism Spectrum Quotient score was created by summing all the items. Total scores range from 0 to 50, with the higher scores indicating more autistic-like traits. A cut-off AQ score of ≥ 32 has been previously determined to identify individuals with clinical levels of ASD symptomatology (Baron-Cohen et al. 2001). The AQ questionnaire has shown acceptable test–retest and inter-rater reliability (Baron-Cohen et al. 2001). For ethical reasons, participants in the Raine Study who had a previously reported diagnosis of ASD were excluded from completing this questionnaire, and therefore were not included in our study.

### Statistical Analysis

To investigate the relationship between the AQ and diet, our analysis considered a range of potential confounding factors. Child characteristics included: gender, gestational age (week of pregnancy), whether they were breastfed, breastfeeding duration, age milk other than breast milk was introduced, and age complementary foods were introduced. Maternal or family characteristics included: family income, maternal education, maternal age (at birth) and mother’s parity (number of previous births, asked at birth) (Meyerkort et al. 2012; Scott et al. 2012). Based on preliminary analysis, those variables significantly associated with food variety were: breastfeeding duration, age milk other than breast milk was introduced, family income, maternal age and maternal education. The age milk other than breast milk was introduced was highly correlated with breastfeeding duration (Spearman’s rho = 0.75). As breastfeeding duration had a stronger association with food variety and AQ score, the age milk other than breast milk was introduced was removed from the analysis. Inclusion of maternal education did not improve the adjusted r-squared, and it was highly correlated with family income (p = 0.001) and maternal age (p = 0.001). Therefore, this variable was removed. Although gender was considered in the model based on the literature, inclusion did not improve the model and it was therefore removed. The final adjusted model included maternal age, family income and breastfeeding duration.

Data were analysed using IBM SPSS Statistics for Windows Version 25.0 (IBM Corp. 2017). Food Variety Scores and EAT scores were analysed as a continuous variable. The AQ score variable was not normally distributed and was divided into quartiles. Data were expressed as mean ± SD, unless otherwise stated. Chi-square tests and one-way ANOVA tests were used to compare the subject characteristics across AQ quartiles. For the dietary assessment data, one-way ANOVA tests were initially used to compare the Food Variety Scores, EAT scores and specific food types across AQ quartiles. Partial eta-squared (${\eta }_{p}^{2}$) measured if the effect size was: small (0.01), medium (0.059) or large (0.138) (Cohen 1988). To adjust for potential confounding factors, a General Linear Model (GLM) test was used to examine associations. Hypotheses for the Food Variety and EAT scores were tested at p ≤ 0.05 significance. Post hoc tests were used to locate significant differences between AQ quartiles. Cohen’s d was used to classify the effect size for the post hoc contrasts as small (0.20), medium (0.50) or large (0.80) (Cohen 1988). The analysis of the 40 specific food types included the Benjamini–Hochberg correction to the subsequent p-values due to the multiple testing.

## Results

Of the 1280 participants who completed the AQ score at the 20 year follow up, 811 participants had available data on all three 24-h recalls at years 1, 2 and 3 and these participants were included in the analysis (Fig. 1). In comparison to Raine Study participants who did not complete all of these assessments, our sample included children of mothers who were significantly older in age, more likely to have completed 12 years of schooling, be married with a higher family income, and to have breastfed their child (p = 0.001 for all) (Supplementary Table 1).

The mean ± SD of total AQ score was 15.1 ± 5.48 (range 1–47), with a median (Q_1_–Q_3_ IQR) score of 14 (11–18). AQ scores were divided into quartiles: quartile 1 included those with a score of ≤ 11 (n = 209); quartile 2 included scores 12–14 (n = 202); quartile 3 included scores 15–18 (n = 213); and quartile 4 included scores ≥ 19 (n = 187).

Subject characteristics across the AQ quartiles are shown in Table 2. Boys were more likely to have an AQ score in the higher range than girls (p = 0.007). In our sample, less than 1 in 5 females scored in the upper quartile, whilst more than 1 in 4 boys scored in this range. There was a significant inverse relationship between AQ quartiles and breastfeeding duration (p = 0.027) with a significant difference between quartile 1 and quartile 4 (p = 0.019). Those in quartile 1 with less autistic-like traits, were breastfed for 9.5 ± 7.63 months, compared to those in quartile 4 with more autistic-like traits, who were breastfed for 7.4 ± 7.02 months. Gestational age, age milk other than breastmilk was introduced, age of introduction to complementary foods, maternal age, mother’s highest school year completed, mother’s parity and family income did not differ significantly across AQ quartiles.

**Table 2:** Comparison of subject characteristics across autism spectrum quotient quartiles

Subject characteristics	Total sample^†^ N (%) or Mean ± SD	Autism spectrum quotient score^‡^				p value
		*Quartile 1* N (%) or Mean ± SD	*Quartile 2* N (%) or Mean ± SD	*Quartile 3* N (%) or Mean ± SD	*Quartile 4* N (%) or Mean ± SD	
Child characteristics						
Male^§^	393 (48.5)	86 (21.9)	94 (23.9)	104 (26.5)	109 (27.7)	0.007*
Female^§^	418 (51.5)	123 (29.4)	108 (25.8)	109 (26.1)	78 (18.7)	
Ever breastfed—Yes^§^	746 (92.2)	192 (25.7)	192 (25.7)	193 (25.9)	169 (22.7)	0.321
Ever breastfed—No^§^	63 (7.8)	16 (25.4)	10 (15.9)	20 (31.7)	17 (27.0)	
Breastfeeding duration (months)^¶^	8.3 ± 7.07	9.5 ± 7.63**	8.1 ± 6.56	8.1 ± 6.90	7.4 ± 7.02**	0.027*
Age milk other than breastmilk was introduced (months)^¶^	4.8 ± 3.59	4.9 ± 3.37	4.9 ± 3.53	5.0 ± 3.77	4.5 ± 3.71	0.479
Age complementary foods introduced (months)^¶^	4.4 ± 1.28	4.5 ± 1.31	4.4 ± 1.24	4.3 ± 1.19	4.6 ± 1.39	0.107
Gestational age (in weeks)^¶^	38.9 ± 1.98	38.7 ± 1.97	39.0 ± 1.45	38.9 ± 1.88	38.7 ± 2.53	0.385
Maternal/family characteristics						
Mean age of mother at birth (years)^¶^	29.3 ± 5.51	29.9 ± 5.26	29.6 ± 5.79	29.1 ± 5.76	28.8 ± 5.12	0.142
Highest school year completed by mother ^§^						
≤ Grade 10	259 (36.2)	61 (23.6)	70 (27.0)	62 (23.9)	66 (25.5)	0.362
Grade 11	97 (13.6)	25 (25.8)	23 (23.7)	32 (33.0)	17 (17.5)	
Grade 12	359 (50.2)	99 (27.6)	80 (22.3)	99 (27.6)	81 (22.5)	
Mothers parity ^¶^	0.9 ± 1.03	0.8 ± 1.03	0.8 ± 1.03	0.9 ± 0.99	0.9 ± 1.08	0.852
Annual Family Income ($AUD) ^§^						
< $23,999	236 (29.6)	45 (19.1)	63 (26.7)	70 (29.6)	58 (24.6)	0.151
$24,000—$35,999	251 (31.5)	69 (27.5)	65 (25.9)	59 (23.5)	58 (23.1)	
> $36,000	310 (38.9)	92 (29.7)	71 (22.9)	80 (25.8)	67 (21.6)	

^*^Significant at p ≤ 0.05

^**^Post hoc association between quartile 1 and quartile 4, p = 0.019

^†^Percentages are expressed relative to the sample total N

^‡^Percentages are expressed relative to the n in each characteristic category

^§^Chi-square test

^¶^One-way ANOVA

Sample size of subject characteristic variables: Gender: n = 811; Did they ever breastfeed: n = 809; Breastfeeding duration: n = 807; Age milk other than breastmilk was introduced: n = 801; Age complementary foods introduced: n = 807; Gestational age (number of weeks of pregnancy from conception to birth): n = 809; Mean age of mother at birth: n = 810; Mother’s highest school year completed: n = 715; Mothers parity (how many times she has previously given birth): n = 796; Annual family income (assessed at year 1): n = 797

Autism Spectrum Quotient (AQ) is a measure of autistic-like traits in the general population and ranges from 0 – 50. A higher score indicates more autistic-like traits. Scores were split into quartiles: quartile 1: scored 0–11 points, quartile 2: scored 12–14 points, quartile 3: scored 15–18 points, and quartile 4: scored 19–50 points

### Dietary scores compared to autism spectrum quotient scores

#### Food variety score

The Food Variety Score was similar across ages 1, 2 and 3 years, with scores of 10.6 ± 2.94, 11.0 ± 2.99 and 11.0 ± 2.82 respectively. Therefore, the three scores were combined to give a mean total food variety score (10.9 ± 2.30). Table 3 presents a comparison of the dietary scores across AQ quartiles. There was a significant downward trend between AQ quartiles 1 and 4 for: total food variety score (p = 0.007, ${\eta }_{p}^{2}$=0.015); core food variety score (p = 0.003, ${\eta }_{p}^{2}$=0.017); and dairy variety score (p = 0.001, ${\eta }_{p}^{2}$=0.021). A similar non-significant downward trend was noted for other Food Variety Scores, except the Meat and Alternatives Variety Score (unadjusted ANOVA).

**Table 3:** Food variety scores and EAT scores across autism spectrum quotient quartiles

Dietary score	Total score N = 811, unadjusted Mean ± SD	Autism spectrum quotient score (mean ± SD)				Unadjusted p value	Partial eta-squared (${\eta }_{p}^{2}$)
		Quartile 1	Quartile 2	Quartile 3	Quartile 4		
Food variety scores							
Total food variety score	10.9 ± 2.30	11.3 ± 2.25^†^	10.9 ± 2.22	10.8 ± 2.25	10.5 ± 2.43^†^	0.007*	0.015
Discretionary variety score	2.83 ± 1.01	2.86 ± 0.99	2.85 ± 0.99	2.80 ± 1.04	2.78 ± 1.01	0.852	0.001
Core food variety score	8.04 ± 1.94	8.42 ± 1.93^†^	8.01 ± 1.90	7.99 ± 1.87	7.70 ± 2.01^†^	0.003*	0.017
Fruit & vegetable variety score	2.94 ± 1.29	3.11 ± 1.32	2.96 ± 1.22	2.88 ± 1.26	2.80 ± 1.33	0.101	0.008
Dairy variety score	1.49 ± 0.52	1.60 ± 0.52^†,‡^	1.46 ± 0.50 ^†^	1.48 ± 0.52	1.39 ± 0.54^‡^	0.001*	0.021
Grain variety score	2.21 ± 0.53	2.27 ± 0.48	2.21 ± 0.54	2.22 ± 0.55	2.14 ± 0.52	0.090	0.008
Meat & alternatives variety score	1.40 ± 0.61	1.44 ± 0.61	1.37 ± 0.61	1.41 ± 0.59	1.37 ± 0.62	0.597	0.002
Total EAT scores							
Year 1	43.5 ± 10.0	45.49 ± 10.08^†,‡^	43.33 ± 10.05	42.53 ± 9.95^†^	42.48 ± 9.82 ^‡^	0.007*	0.015
Year 2	40.2 ± 10.2	41.32 ± 9.82^†^	41.36 ± 10.11^‡^	39.93 ± 10.32	38.13 ± 10.37^†,‡^	0.005*	0.016
Year 3	38.3 ± 10.1	40.04 ± 10.47^†^	38.06 ± 10.16	37.41 ± 9.73^†^	37.54 ± 10.08	0.030*	0.011

*Significant at p ≤ 0.05

Partial eta-squared (${\eta }_{p}^{2}$) measures effect size: small (0.01), medium (0.059), large (0.138).

^†,‡^Quartiles with the same superscript number were significantly different in post hoc analysis (p ≤ 0.05, Tukey).

Autism Spectrum Quotient (AQ) score: measures autistic-like traits in the general population and ranges from 0 – 50. A higher score indicates more autistic-like traits. Scores were split into quartiles: quartile 1: scored 0-11 points, quartile 2: scored 12-14 points, quartile 3: scored 15-18 points, and quartile 4: scored 19-50 points.

Food Variety Score: measures the number of different food types eaten on a daily basis. Data for ages 1, 2 and 3 were combined to give a mean score for each variety group. Scores range from 0 to 40. The division of food types are shown in Table 1.

The Raine Eating Assessment in Toddlers (EAT) Score: assessed the quality of the child’s diet at age 1, 2 and 3. The higher the EAT score, the better diet quality. Scores range from 0 to 70.

Unadjusted p value: confounding factors were not taken into account. One-way ANOVA test used.

**Fig. 2:**
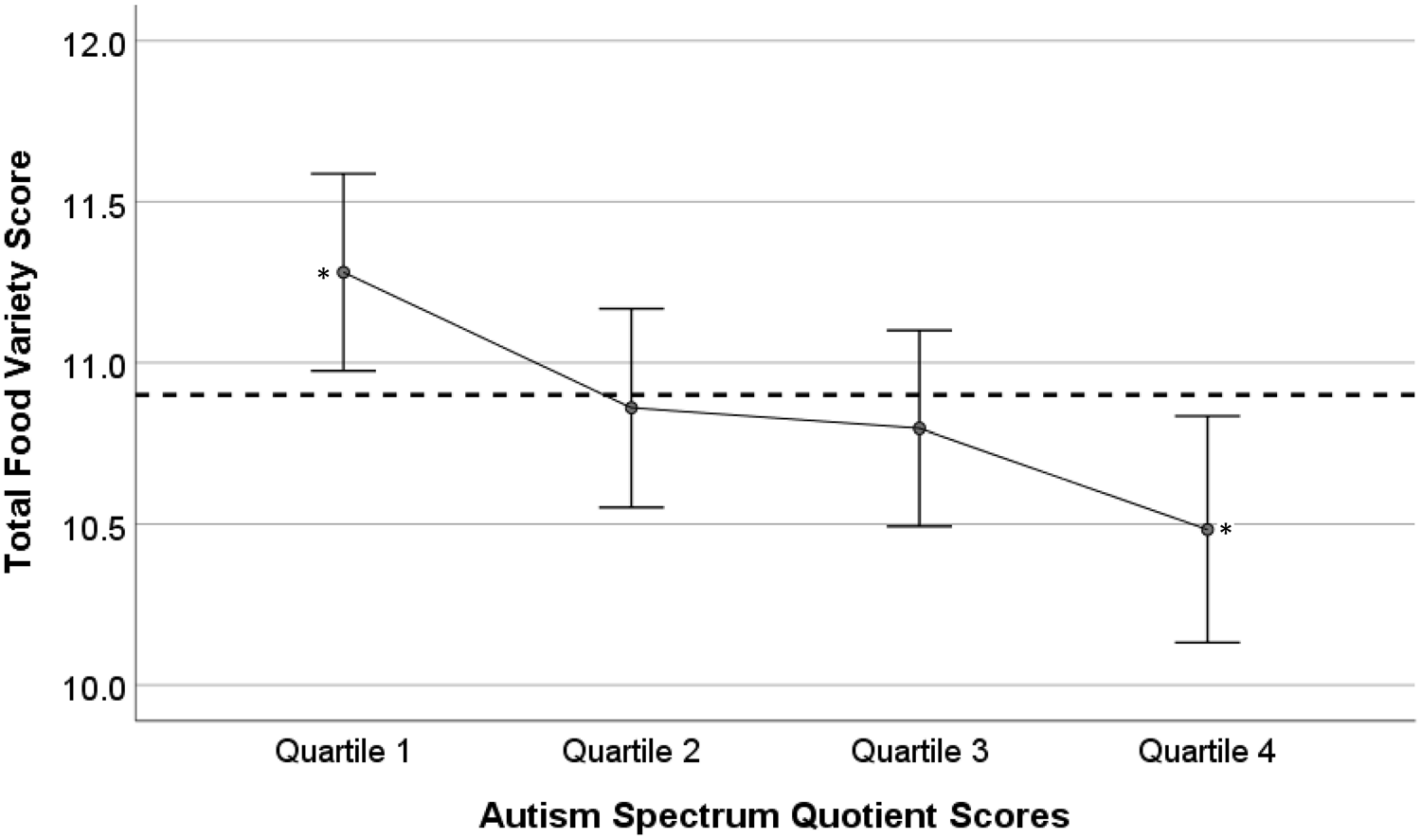
Comparison of total food variety scores and autism spectrum quotient scores across quartiles (unadjusted). *Significant post-hoc differences at p ≤ 0.05. The dotted line represents the mean Total Food Variety Score of children in the study. Error bars represent 95% Confidence Interval. Total Food Variety Score: measures the number of different food types eaten on a daily basis. Data for ages 1, 2 and 3 were combined to give a mean score for each variety group. Scores range from 0 to 40. The division of food types are shown in Table 1. Autism Spectrum Quotient (AQ): is a measure of autistic-like traits in the general population and ranges from 0 to 50. A higher score indicates more autistic-like traits. Scores were split into quartiles: quartile 1: scored 0–11 points, quartile 2: scored 12–14 points, quartile 3: scored 15–18 points, and quartile 4: scored 19–50 points. Reference Baron-Cohen et al. (2001)

Figure 2 shows a means plot of Total Food Variety scores compared to the AQ scores across quartiles. Based on the trend observed in this plot, there was only a marginal difference between quartiles 2 and 3, and to the overall mean. However, the difference between quartiles 1 and 4 was profound, along with their comparison to the overall mean, and the significance of this is of interest. Therefore, in our subsequent GLM, we explored the contrasts between quartile 1 and 4, quartile 1 and overall mean, and quartile 4 and overall mean.

Adjusted analyses using GLM showed that children in AQ quartile 1 consumed significantly greater variety in Total Food (p = 0.027, d = 0.18), Core Food (p = 0.021, d = 0.19), and Dairy (p = 0.009, d = 0.24) compared to those in quartile 4 (Table 4). The same differences were observed between those in quartile 1 and the overall mean of children in the study (all p < 0.05 and d < 0.23). Furthermore, the children in quartile 4 consumed significantly lower variety in Total Food (p = 0.048, d = − 0.14), Core Food (p = 0.047, d = − 0.15), and Dairy (p = 0.035, d = − 0.16) compared to the overall mean. Supplementary Fig. 1 shows a comparison of an adjusted means plot of the Food Variety Scores compared to the AQ score quartiles and illustrates the downward trends between quartile 1 and quartile 4.

**Table 4:** Contrast results of food variety scores and EAT scores across Autism Spectrum Quotient quartiles

Dietary score	Contrast	Mean difference (95% CI)	p-value	p-value^†^	Cohen’s d
Total food variety	AQ quartile 1 vs AQ quartile 4	0.6 (0.1, 1.0)	0.012*	0.027*	0.18
	AQ1 vs mean total food variety	0.3 (0.0, 0.6)	0.032*	0.041*	0.15
	AQ4 vs mean total food variety	-0.3 (-0.6, 0.0)	0.048*	0.048*	-0.14
Core food variety	AQ quartile 1 vs AQ quartile 4	0.5 (0.1, 0.9)	0.007*	0.021*	0.19
	AQ1 vs mean core food variety	0.3 (0.1, 0.5)	0.015*	0.027*	0.17
	AQ4 vs mean core food variety	-0.2 (-0.5, 0.0)	0.042*	0.047*	-0.15
Dairy food variety	AQ quartile 1 vs AQ quartile 4	0.2 (0.1, 0.3)	0.001*	0.009*	0.24
	AQ1 vs mean dairy food variety	0.1 (0.0, 0.2)	0.002*	0.009*	0.23
	AQ4 vs mean dairy food variety	-0.1 (-0.1, 0.0)	0.023*	0.035*	-0.16
EAT score year 1	AQ quartile 1 vs AQ quartile 4	2.1 (0.2, 4.1)	0.032*	0.058	0.15
	AQ1 vs mean at year 1	1.6 (0.4, 2.7)	0.008*	0.024*	0.19
	AQ4 vs mean at year 1	-0.5 (-1.7, 0.7)	0.379	0.426	-0.06
EAT score year 2	AQ quartile 1 vs AQ quartile 4	2.7 (0.7, 4.7)	0.008*	0.024*	0.19
	AQ1 vs mean at year 2	0.9 (-0.3, 2.1)	0.142	0.183	0.10
	AQ4 vs mean at year 2	-1.8 (-3.1, -0.6)	0.005*	0.024*	-0.20
EAT score year 3	AQ quartile 1 vs AQ quartile 4	1.8 (-0.2, 3.8)	0.081	0.122	0.12
	AQ1 vs mean at year 3	1.4 (0.2, 2.6)	0.020*	0.045*	0.17
	AQ4 vs mean at year 3	-0.4 (-1.6, 0.9)	0.577	0.577	-0.04

*Significant at p ≤ 0.05

^†^Benjamini–Hochberg correction was applied

Contrast results examined through General Linear Modellings

Cohen’s d effect size: small (0.20), medium (0.50) or large (0.80) (Cohen, 1988)

Autism Spectrum Quotient (AQ): measures autistic-like traits in the general population and ranges from 0 to 50. A higher score indicates more autistic-like traits. Scores were split into quartiles: quartile 1: scored 0–11 points, quartile 2: scored 12–14 points, quartile 3: scored 15–18 points, and quartile 4: scored 19–50 points

Food Variety Score: measures the number of different food types eaten on a daily basis. Data for ages 1, 2 and 3 were combined to give a mean score for each variety group. Scores range from 0 to 40. The division of food types are shown in Table 1

The Raine Eating Assessment in Toddlers (EAT) Score: assessed the quality of the child’s diet at age 1, 2 and 3. The higher the EAT score, the higher the quality in the diet. Scores range from 0 to 70

Examination of the 40 different food types within the Food Variety Score (Table 1), was conducted to ascertain if certain foods were significantly associated with AQ quartiles. A significant inverse association was observed in frequency of consumption of yoghurt (p = 0.04, ${\eta }_{p}^{2}$ = 0.018) and citrus fruits (p = 0.04, ${\eta }_{p}^{2}$ = 0.019) (one-way ANOVA with Benjamini–Hochberg correction). For yoghurt, participants had significantly higher consumption in quartile 1 compared with quartile 4 (p = 0.019). For citrus fruit, consumption was significantly higher in quartile 1 compared to quartiles 3 (p = 0.004) and 4 (p = 0.002). For yoghurt there was a 35% reduction in frequency of consumption from quartile 1 to quartile 4. For citrus fruits, there was a 50% reduction between quartile 1 and quartile 4. No significant differences were observed with other food types (Supplementary Table 2).

#### EAT Scores

As the mean EAT scores differed across years 1, 2 and 3 (Table 3), each year was analysed separately. The EAT scores were normally distributed and compared to the AQ scores across quartiles. There were significant differences between AQ quartiles for: year 1 (p = 0.007, ${\eta }_{p}^{2}$ = 0.015); year 2 (p = 0.005, ${\eta }_{p}^{2}$ = 0.016); and year 3 (p = 0.030, ${\eta }_{p}^{2}$= 0.011) (unadjusted ANOVA).

After adjusting for confounding factors, children in quartile 1 had significantly greater diet quality at year 1 (p = 0.024, d = 0.19) and year 3 (p = 0.045, d = 0.17), compared to the overall mean of the children in the study (Table 4). At year 2, children in quartile 4 had significantly lower diet quality compared to quartile 1 (p = 0.024, d = 0.19), and compared to the overall mean (p = 0.024, p = − 0.20) (General Linear Modelling).

## Discussion

To our knowledge, this is the first study to investigate the longitudinal association between self-reported AQ scores in early adulthood, and parent-reported food variety in early childhood. Results showed a modest but significant association between higher autistic-like traits in adulthood and lower food variety and diet quality in childhood (Tables 3 and 4).

Our study showed that as autistic-like traits increased, total food variety, core food variety and dairy variety significantly decreased. When compared to the overall mean score for children in the study, those with less autistic-like traits had significantly greater variety in total food, core food and dairy food groups and those with more autistic-like traits had significantly lower variety in total food, core foods and dairy food groups (Table 4). In addition, those with higher autistic-like traits had significantly lower frequency of consumption of specific foods including yoghurt and citrus fruits.

Although no previous studies have looked at autistic-like traits and food variety, a number of studies have examined food variety in children with diagnosed ASD. Findings from these studies are similar to our results. Children with ASD compared to typically developing children have been shown to eat less types of foods (19 foods versus 23 foods) (Chistol et al. 2018) and fewer foods per month (33.5 versus 54.5) (Zimmer et al. 2012). Factors that may contribute to lower food variety in children with ASD include restrictive and repetitive behaviours, behavioural inflexibility (Suarez et al. 2014a, b), sensory sensitivities and gastrointestinal problems (Kuschner et al. 2015). These issues affect food intake, and therefore could also contribute to the low food variety observed in childhood, of people with higher autistic-like traits.

It is possible that diet could affect the severity of autistic-like traits. Restrictive feeding issues present in a child with ASD may affect their cognitive, motor and behavioural development (Ranjan and Nasser 2015)*.* This in turn may impact upon their abilities to communicate. For example, low iron can affect brain metabolism and impair cognition by altering neurotransmitter synthesis, decreasing production of myelin and the function of basal ganglia and impairing synaptogenesis (Pivina et al. 2019). Therefore, a child with ASD who is also low in iron may find it more difficult to communicate and behave in a socially acceptable manner (Ranjan and Nasser 2015). Diet is also a powerful modulator for influencing the composition of the gut microbiome (Tomova et al. 2020). Emerging evidence suggests that nutritional intervention in children with ASD may improve immune status and GI function by altering the intestinal microbiota (Tomova et al. 2020). More research is required to investigate improvements in autistic-like traits with dietary intervention.

A notable result in our study was that young adults with higher autistic-like traits were lower consumers of dairy, particularly yoghurt, in early childhood. Diets low in dairy products are commonly noted in children with ASD (Marshall et al. 2014; Neumeyer et al. 2018; Schreck et al. 2004), for example one study reported that children with ASD had 2.2 serves of dairy per day compared to 3.4 serves in typically developing children (Herndon et al. 2009). Dairy is an important source of calcium that is essential for optimal bone development (Graf-Myles et al. 2013). Lower intakes of dairy may be explained by some children with ASD following a gluten-free casein-free diet to treat ASD symptoms (Diolordi et al. 2014; Herndon et al. 2009; Kral et al. 2013; Sharp et al. 2013). This diet excludes the proteins gluten, found in wheat, rye and barley, and casein found in dairy products, although there is no conclusive evidence that ASD symptoms improve on this diet (Graf-Myles et al. 2013; Sathe et al. 2017). The participants in our study were in the general non-ASD population and therefore unlikely to be exposed to popular ASD diets, yet those with higher autistic-like traits still had low dairy consumption. This provides important and unbiased information that dairy intake, and in particular the nutrient calcium, could be of concern in this population.

The Core Food Variety Score results showed that adults with more autistic-like traits were significantly more likely to have had lower intakes of healthy foods from the five core food groups in childhood. Previous studies have identified that children with ASD have low fruit and vegetable intake (Chistol et al. 2018; Emond et al. 2010; Marshall et al. 2014; Schreck et al. 2004; Sharp et al. 2013), and we identified low citrus intake specifically in those with higher autistic-like traits. However, some studies have found that children with ASD eat more fruit than those without ASD (Esteban-Figuerola et al. 2019; Herndon et al. 2009), while other studies reported no difference in fruit and vegetable intake between groups (Graf-Myles et al. 2013). Research has reported that children with ASD may also eat more processed or unhealthy snack foods containing more fat and sugar (Cermak et al. 2010; Sharp et al. 2013). However, our study showed that those with higher autistic-like traits did not consume a greater variety of discretionary items in early childhood. Comparison of our results to the literature is conflicting, likely due to the diverse and multifactorial nature of ASD. Further studies are needed to confirm these results.

The food variety results were supported by our subsequent analysis of diet quality. Our results showed higher autistic-like traits in early adulthood were associated with lower EAT scores at years 1, 2 and 3, representing diet quality. Compared to the average score for children in the study, those with less autistic-like traits, had significantly greater diet quality at years 1 and 3, and those who had higher autistic-like traits had significantly lower diet quality at year 2 (Table 4). In addition, those at year 2 showed that as autistic-like traits increased, their diet quality significantly decreased. Results of previous studies on diet quality in children with ASD are conflicting. In a review article that examined diet quality among children with ASD, results were inconsistent with regard to nutritional adequacy (Kral et al. 2013), potentially due to different dietary assessment methods used. Authors concluded that children with ASD with more food selectivity and less food variety were more likely to have inadequate nutrient intakes (Kral et al. 2013). A study using a Healthy Eating Index to measure diet quality, found no difference between the scores of children with ASD and those without (Graf-Myles et al. 2013). Similarly, another article reported no significant differences in diet quality between children with ASD and those without (Marí-Bauset et al. 2017).

It is concerning that children with ASD are not routinely screened for feeding problems (Ranjan and Nasser 2015). Whilst picky eating is considered common in toddlers, children with ASD often experience more severe and longer-term feeding difficulties, and parents sometimes require extensive support (Rogers et al. 2012). Picky eating has been reported to be more prominent among children with higher autistic-like traits. In a population-based cohort study, children with high autistic-like traits at six years of age possessed more picky eating behaviours at ten years of age (van 't Hof et al. 2020). Feeding difficulties can be a source of stress for families and severely impact the family’s quality of life (Suarez et al. 2014a, b). Our study showed that although the difference was small, those with higher autistic-like traits had childhood diets that were significantly lower in food variety and diet quality. Improving the nutritional status and addressing feeding difficulties in children who display autistic-like traits from an early age, regardless of ASD diagnosis, should be a priority intervention for the overall health care of this group.

Our study utilised a longitudinal cohort with a relatively large sample size. The ability to investigate AQ results in adulthood and compare with data previously collected in early childhood is a remarkable aspect of the Raine Study. A potential limitation of this study was that the dietary assessment at years 1, 2 and 3 was conducted using a 24-h recall completed by the primary caregiver. Accurate quantification of consumption amounts can be difficult in early childhood due to children learning to eat and some food being dropped rather than consumed. The use of one day rather than a longer time period may have limited the ability to accurately assess their usual diet. To address this issue, the mean Food Variety Scores were combined for years 1, 2 and 3 to provide an average score of food variety over this time.

Our study group had mothers who were significantly older in age, had a higher education level, a higher family income, and were more likely to be married, compared to other participants of the Raine Study who did not complete the diet and AQ components. Therefore, further studies are needed to confirm trends in a more representative sample. However, it is important to note that in the Raine Study, socially disadvantaged families were in the majority for initial recruitment, which may make the findings of our study more generalizable. Further, data from the Avon Longitudinal Study of Parents and Children in the United Kingdom suggest that selective dropout in cohort studies may only marginally affect results (Wolke et al. 2009).

It is currently unknown whether dietary variety and quality in childhood improves over time in people who have more autistic-like traits. This is an important area for future investigation. Low dairy consumption in children in ASD is frequently reported in the literature and was also noted in our study. Further investigation into calcium consumption and bone mineral density is warranted, as well as further analysis into the possible link, and direction of association, between breastfeeding duration and autistic-like traits. We propose the introduction of a screening tool to assess feeding problems in those children with high autistic-like traits. This could be utilised during the early investigative stages of an ASD diagnosis. Those not meeting the criteria for ASD, but still possessing a high-level of autistic-like traits, could be referred to a dietitian for further investigation of their diet.

Our results suggest that young adults with higher autistic-like traits were more likely to have a modest reduction in food variety and quality in early childhood, with particularly low intakes of dairy and citrus. Our results highlight the need to provide nutritional assistance to families who have children displaying autistic-like traits at a young age. Whilst we know that children with ASD are a nutritionally vulnerable group, those who have higher levels of autistic-like traits may also be vulnerable. Children with more autistic-like traits could benefit from dietary intervention to ensure they have the best chance to grow and reach their full developmental potential.

## Electronic supplementary material

Below is the link to the electronic supplementary material.

Supplementary file 1 (DOCX 278 kb)
