# Supplementary material for: Young Adults with High Autistic-Like Traits Displayed Lower Food Variety and Diet Quality in Childhood
Source: J Autism Dev Disord. 2020 Jul 2;51(2):685–96. doi: 10.1007/s10803-020-04567-4 (PMC7835288; doi:10.1007/s10803-020-04567-4)
Supplement: Supplementary file 1 — (DOCX 278 kb) [file 10803_2020_4567_MOESM1_ESM.docx]

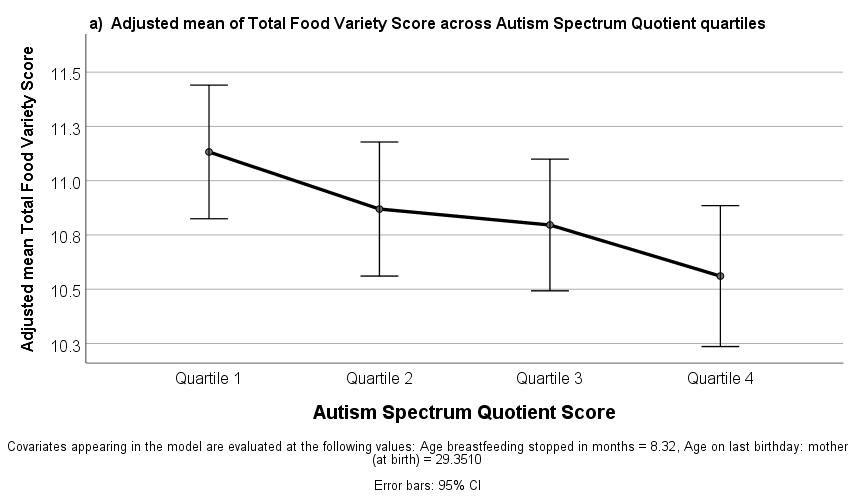

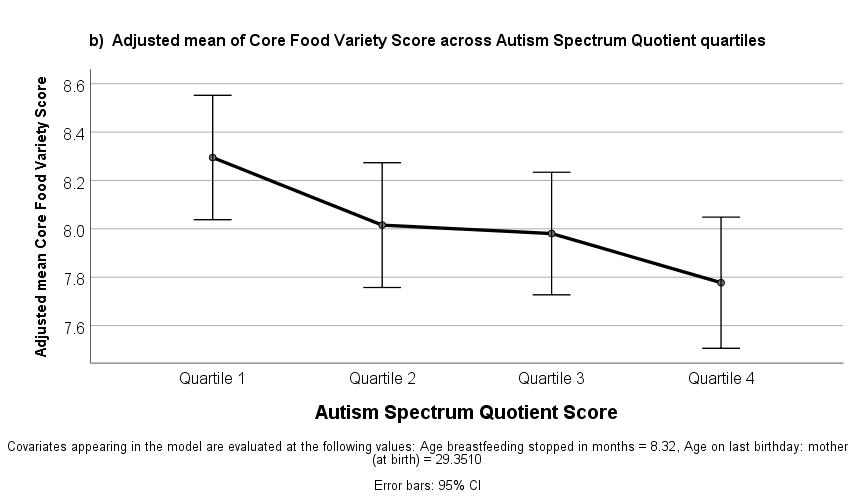


*

*

*

*


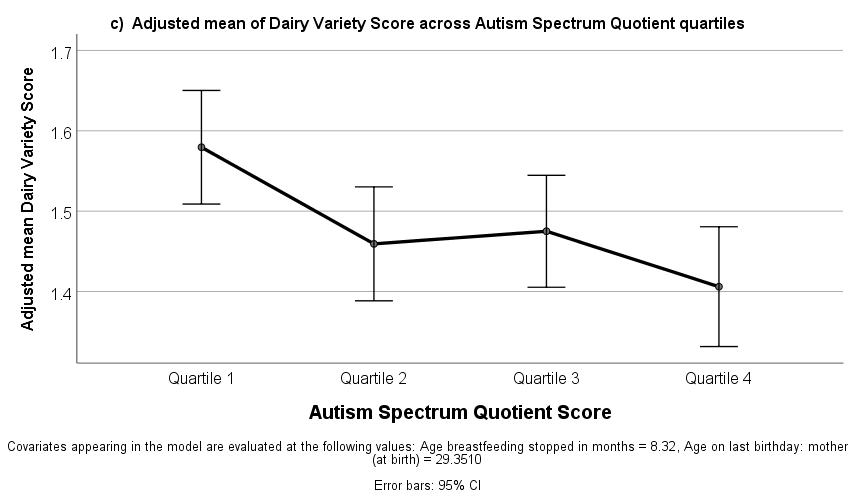


**SUPPLEMENTARY FIGURE 1*.***

Adjusted means plots comparing dietary scores and AQ scores across quartiles

* Significant differences located between quartiles 1 and 4 (p ≤ 0.05).

Autism Spectrum Quotient (AQ) Score: measures autistic-like-traits in the general population, with a higher score indicating more autistic-like-traits. Quartile 1: scored 0-11 points, quartile 2: scored 12-14 points, quartile 3: scored 15-18 points, and quartile 4: scored 19-50 points.

Food Variety Scores: measures the number of different food types eaten on a daily basis. Data for ages 1, 2 and 3 were combined to give a mean score for each variety group. Scores range from 0 to 40. The definitions and division of scores are shown in Table 1.

The Raine Eating Assessment in Toddlers (EAT) Score: assessed the quality of the child’s diet at age 1, 2 and 3. The higher the EAT score, the higher the quality in the diet. Scores range from 0 to 70.

*

*


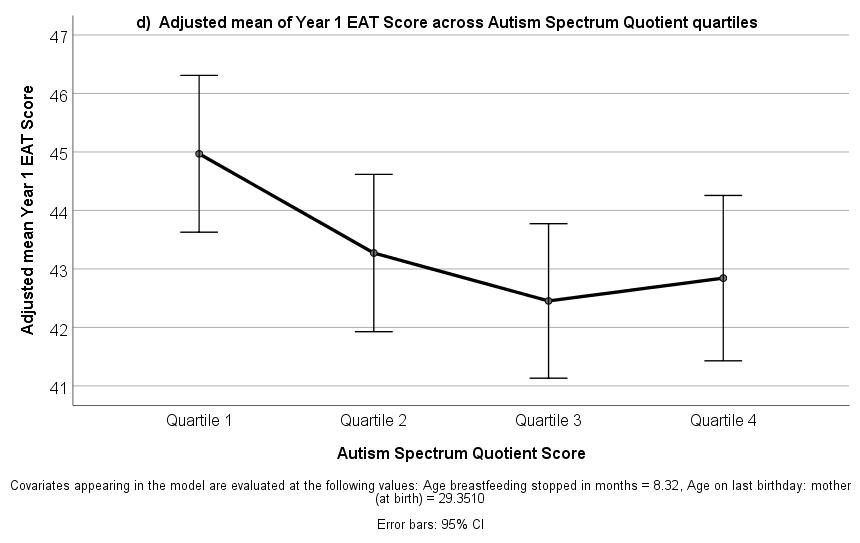

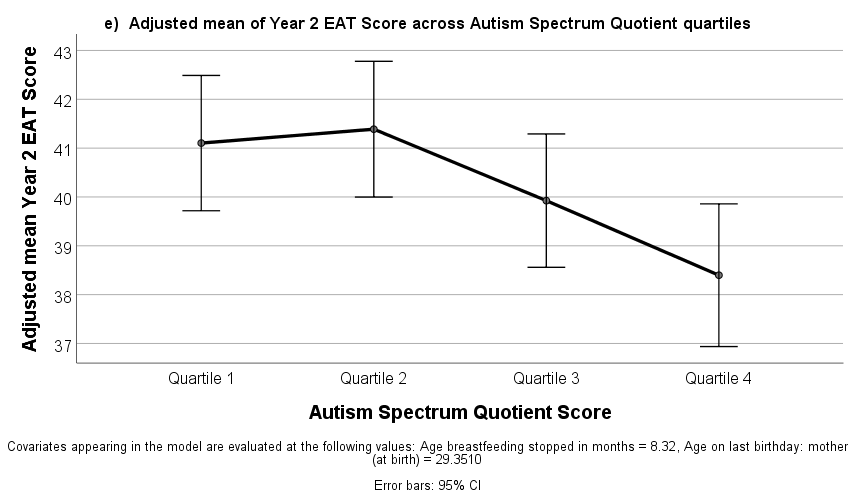


*

*


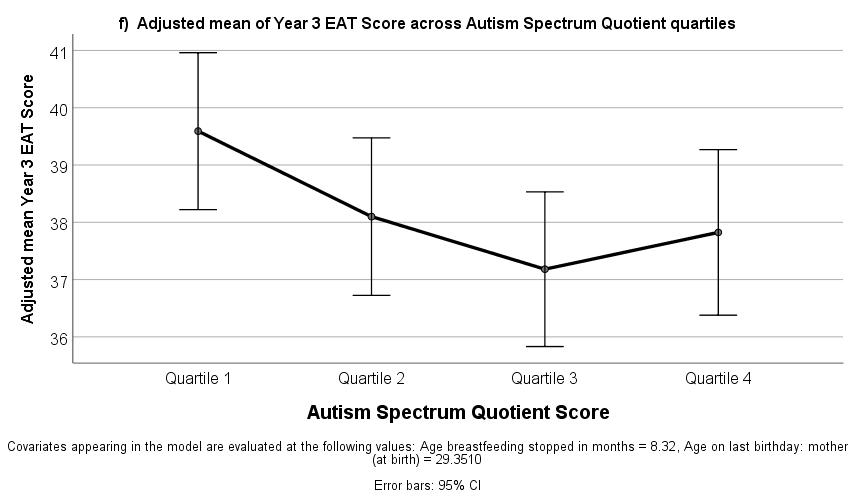


**SUPPLEMENTARY TABLE 1**

Characteristics of Raine Study participants who were included in our study compared to those who were not included (withdrawn, deceased, or relevant assessments not completed).

| **Characteristics** | **Total overall subjects in the Raine study N=2868** | | | |
| --- | --- | --- | --- | --- |
|  | **Included in this study N=811** | **Excluded from this study**  **N=2057** | | **P** |
|  | **N (valid %)** | | **N (valid %)** |  |
| **Child Characteristics**  Male  Female | 393 (48.5)  418 (51.5) | | 1061 (51.6)  996 (48.4) | 0.132 |
| Breastfeeding - Yes  Breastfeeding - No  Breastfeeding - not stated | 746 (92.2)  63 (7.8)  2 | | 1408 (87.9)  193 (12.1)  34 | 0.001 * |
| **Maternal/ Family Characteristics**  Mean age of mother at birth (years)    Highest School Year Completed  ≤ Grade 10  Grade 11  Grade 12  Missing | 29.3 (N=810)  259 (36.2)  97 (13.6)  359 (50.2)  96 | | 26.9 (N=2051)  775 (49.3)  219 (14.0)  577 (36.7)  486 | 0.001 *  0.001 * |
| Annual Family Income at birth ($AUD)  < $23,999  $24,000 - $35,999  > $36,000  Missing | 236 (29.6)  251 (31.5)  310 (38.9)  14 | | 710 (46.7)  416 (27.4)  393 (25.9)  538 | 0.001 * |
| Parental marital status  Not Married (never, separated, divorced or widowed)  Married or de facto relationship  Missing | 77 (9.7)  719 (90.3)  15 | | 390 (19.4)  1618 (80.6)  49 | 0.001 * |

* Significant at p ≤ 0.05.

**SUPPLEMENTARY TABLE 2**

Data Analysis for 40 Food Types was conducted across Autism Spectrum Quotient quartiles, sorted by significance

| **Food Items** | **Unadjusted p** | **Adjusted p** ^†^ |
| --- | --- | --- |
| Citrus fruits | 0.001 * | 0.04 * |
| Yoghurt | 0.002 * | 0.04 * |
| Sugar (added & other desserts) | 0.024 * | 0.24 |
| Cheese | 0.029 * | 0.24 |
| Other fruits | 0.03 * | 0.24 |
| Cereal/Granola bars | 0.044 * | 0.29 |
| Carbonated drinks | 0.112 | 0.61 |
| Milk desserts | 0.121 | 0.61 |
| Dried fruit | 0.185 | 0.72 |
| Other meats e.g. offal | 0.187 | 0.72 |
| Cruciferous vegetables | 0.213 | 0.72 |
| Salty snacks | 0.233 | 0.72 |
| Nuts and Seeds | 0.242 | 0.72 |
| Milk | 0.251 | 0.72 |
| Cakes, pies, cookies, pastries | 0.281 | 0.75 |
| Fish and shellfish | 0.315 | 0.75 |
| Breads and rolls | 0.320 | 0.75 |
| Apples | 0.342 | 0.76 |
| Chicken/turkey | 0.396 | 0.80 |
| Red meat | 0.399 | 0.80 |
| Confectionery | 0.469 | 0.89 |
| Rice and pasta | 0.549 | 0.90 |
| Crackers/pretzels/rice cakes/rusks | 0.571 | 0.90 |
| Pizza/savoury pies & pastries | 0.571 | 0.90 |
| Green Vegetables | 0.605 | 0.90 |
| Unrefined breakfast cereals | 0.622 | 0.90 |
| Legumes & other veg substitutes | 0.623 | 0.90 |
| Summer fruits | 0.633 | 0.90 |
| Hot chips/French fries | 0.670 | 0.91 |
| Potatoes | 0.712 | 0.91 |
| Tomatoes | 0.718 | 0.91 |
| Pears | 0.755 | 0.91 |
| Refined breakfast cereals | 0.777 | 0.91 |
| Hotdogs, sausages, deli meats | 0.783 | 0.91 |
| Eggs | 0.796 | 0.91 |
| Yellow/orange vegetables | 0.827 | 0.91 |
| Other vegetables | 0.838 | 0.91 |
| Fruit-sweetened drinks | 0.880 | 0.91 |
| Bananas | 0.891 | 0.91 |
| Added fats and oils | 0.916 | 0.92 |

^†^ Benjamini-Hochberg correction is applied.
* Significant at p ≤ 0.05.

See complete description on each food type on Table 1.
